# Cluster Headache and Migraine Shared and Unique Insights: Neurophysiological Implications, Neuroimaging, and Biomarkers: A Comprehensive Review

**DOI:** 10.3390/jcm14072160

**Published:** 2025-03-21

**Authors:** Gabriele Bertotti, Vicente Fernández-Ruiz, Alberto Roldán-Ruiz, Miguel López-Moreno

**Affiliations:** 1 Departamento de Fisioterapia, Facultad de Ciencias de la Salud, Universidad Francisco de Vitoria, Ctra. Pozuelo-Majadahonda Km 1,800, Pozuelo de Alarcón, 28223 Madrid, Spain; gabriele.bertotti@ufv.es (G.B.); vicente.fernandez@ufv.es (V.F.-R.); miguel.lopez@ufv.es (M.L.-M.); 2 CranioSPain Research Group, Centro Superior de Estudios Universitarios La Salle, Universidad Autónoma de Madrid, 28023 Madrid, Spain

**Keywords:** cluster headache, migraine, neurophysiology, pathophysiology, neuroimaging, biomarker, chronic pain

## Abstract

Migraine headache (MH) and cluster headache (CH) are debilitating primary headache disorders that impose a significant global burden. While they share certain clinical features, such as unilateral pain and autonomic dysfunction, their underlying pathophysiological mechanisms remain distinct. Advances in the understanding of neurophysiological features, such as neuroimaging and biomarker research, have provided critical insights into both their overlapping and divergent characteristics. Neurophysiological research has revealed differences in nociceptive processing, cortical excitability, and sensory integration, underscoring the complexity of these conditions. Neuroimaging studies reveal common activation patterns within pain-processing networks, including the trigeminal system and hypothalamus, while highlighting key differences, such as hypothalamic hyperactivity in CH and cortical alterations in MH. Additionally, biomarker research has identified shared elements, including elevated calcitonin gene-related peptide (CGRP), yet distinct variations in its regulation and genetic predispositions. Genome-wide association studies have further elucidated the genetic architecture of these disorders, uncovering susceptibility loci that reinforces their unique yet occasionally intersecting genetic foundations. These multifield advancements not only enhance the understanding of MH and CH pathophysiology but also pave the way for improved diagnostic precision, personalized therapeutic strategies, and future research.

## 1. Introduction

Cluster headache (CH) and migraine headache (MH) are among the most disabling primary headache disorders, significantly impacting patients’ quality of life and healthcare utilization [1]. The global prevalence of MH is estimated at 15%, with a higher burden among women, whereas CH, though rarer (affecting approximately 0.1% of the population), is more prevalent in men and often described as one of the most excruciating pain conditions [2,3]. Both disorders contribute to substantial disability, with MH ranked as the second leading cause of years lived with disability worldwide [4], while CH, due to its severe nature, is associated with increased psychiatric comorbidities and even suicidal ideation [5].

Beyond individual suffering, these headache disorders impose a notable economic burden. In Europe, migraine-related costs exceed EUR 100 billion annually, driven by productivity losses and healthcare expenditures [6]. Similarly, CH, despite its lower prevalence, leads to significant direct and indirect costs due to frequent healthcare visits, medication use, and reduced work performance [7]. In this regard, early and effective management is critical. Non-pharmacological approaches, including structured headache education, lifestyle modifications, and neurostimulation techniques, are increasingly recognized as adjunctive strategies that may reduce healthcare costs while improving patient outcomes [8,9].

CH and MH share overlapping features, which can contribute to misdiagnosis and delays in appropriate treatment. Both conditions manifest with unilateral severe head pain, and although CH typically presents autonomic symptoms, MH can also exhibit this autonomic pattern. Similarly, classic MH symptoms include photophobia, phonophobia and nausea, but CH can also report sensitivity to light and sound or mild nausea. Although the last version of the International Classification of Headache Disorders (ICHD-III) clearly differentiates CH from MH [10], none of the headache features are specific to any headache diagnosis [11]. These overlapping features are clinically and socioeconomically relevant, since they can lead to delays in appropriate diagnosis and treatment.

In light of these considerations, this narrative review aims to address a highly relevant topic: understanding shared insights in CH and MH by exposing overlapping and unique neurophysiological characteristics, as well as neuroimaging and biomarking features of these two conditions.

Specifically, we hypothesize that CH and MH share common neurophysiological mechanisms while also presenting distinct pathophysiological features that may help refine their differential diagnosis.

## 2. Neurophysiological Implications in Cluster Headaches and Migraines

### 2.1. Pathophysiological Mechanisms

Pathophysiological characteristics of these two conditions include both peripheral and central mechanisms.

First, both in CH and MH patients, the trigeminovascular system plays a key role in the unilateral distribution of pain, as the activation of this system leads to the release of neuropeptides (calcitonin gene-related peptide (CGRP), nitric oxide synthase, pituitary adenylate cyclase-activating polypeptide-38 (PACAP-38), substance P, and neurokinin A) from the sensory nerve terminals of the trigeminal nerve [12]. For a long time, it was considered the most important factor, as, in humans, activation of meningeal and vascular afferents produces headache, a mechanism traditionally considered of peripheral origin [13]. However, it is now known that any nociceptive input triggers activations of central structures related to pain processing, which, along with the convergence of trigeminal and cervical nociceptive afferents in the caudal portion of the spinal trigeminal nucleus, explains that pain in CH and in MH is more attributed to central rather than peripheral changes [12]. For instance, since the 1970s, the theory of cavernous sinus inflammation could no longer explain the onset of a CH attack [14], highlighting that there had been the necessity of this paradigm shift in understanding pain for many years already. Like in MH, the phenomenon of vasodilation that, according to the previous paradigm, explained the origin of symptoms, is now considered to create a permissive state in the brain of CH patients [3]. Thus, it is understandable that, on one hand, vasodilation may represent one of the possible triggers, but on the other hand, it should not be considered the cause of the disease, but rather an epiphenomenon [14].

Alternatively, around 30% of MH patients experience aura symptoms during their attacks. Cortical spreading depression (CSD), a wave of cortical depolarization followed by neuronal suppression, is widely recognized as the underlying mechanism of aura [15]. Schulte et al. (2016), after observing a MH patient without aura for 30 consecutive days, identified altered functional connectivity between the hypothalamus, the spinal trigeminal nuclei, and the dorsal pons during both the preictal and pain phases [14]. The authors suggested that these functional changes within the network could be the primary trigger of MH attacks [16]. Moreover, recent research has suggested a potential role of the cerebellum in MH pathophysiology, as trigeminal pain stimulation in patients during a MH attack has shown coactivation of the cerebellum and periaqueductal gray (PAG) [17]. In general, multiple monoaminergic and peptidergic systems are involved in MH pathogenesis, some of which play a role in both pain and other migraine-associated symptoms. While not all answers are known, imaging studies during the premonitory phase strongly suggest that the vascular and neuroinflammatory theories alone cannot fully explain the clinical phenotype of MH or the brain changes observed in imaging. It is evident that central neuronal mechanisms within complex and overlapping sensory and physiological systems are active early in the attack [18].

### 2.2. The Paradigm Shift

Since the 1990s, sensory neuroplasticity and the identification of psychosocial factors associated with persistent pain have enabled a better understanding of all the determinants involved in the painful experience, marking a progressive departure from the biomedical model that equates pain with tissue damage. This paradigm shift toward the neuroscience of pain has allowed for a better definition of the pathogenic mechanisms of many persistent pain conditions [19], one of which is central sensitization.

Woolf et al. (1988) introduced the term central sensitization in 1988 after observing, through basic research, that hypersensitivity to pain occurs due to changes in neuronal activity at both the peripheral and spinal cord levels [20,21]. Central sensitization has been redefined over the past four decades, and in 2008, the International Association for the Study of Pain (IASP) defined it as a neurophysiological process characterized by an increase in the responsiveness of nociceptive neurons in the central nervous system to normal or subthreshold afferent input [22]. Central sensitization is a dynamic phenomenon that can occur both adaptively and maladaptively [23]. It includes neurobiological changes in the spinal dorsal horn such as neuronal hyperexcitability, increased synaptic efficacy, and reduced inhibition [19]. Changes in synaptic efficacy are explained by modifications in the expression and function of membrane proteins (e.g., ion channels) and in the neuronal structure itself, a phenomenon known as neuroplasticity [24]. These phenomena are not exclusive to the spinal cord but also occur at the supraspinal level [25,26] and appear to be enhanced by cognitive-emotional factors [23]. Additionally, several cortical and subcortical structures can amplify the sensitization characterizing the spinal dorsal horn through “top-down” mechanisms, either by increasing the activity of facilitatory descending pathways or by inhibiting the activity of inhibitory descending pathways [19]. In this regard, the literature indicates that patients with conditions compatible with central sensitization exhibit impaired functioning of the pain-inhibitory descending pathways [27,28].

Although central sensitization cannot be directly measured in humans and thus it cannot be concluded that chronic pain is caused by central sensitization [29], quantitative sensory tests (QST) allow for the measurement of variables that can be considered clinical features suggesting the presence of central sensitization, such as allodynia, secondary hyperalgesia, and temporal summation of pain [30,31]. Central sensitization signs have been consistently reported when describing the somatosensory profile of patients with CH [32] and MH [33].

Several neurophysiological investigations [34,35,36] and clinical studies [37,38] have suggested that patients with CH exhibit changes in pain processing in both trigeminal and extratrigeminal areas. This has been recently confirmed by meta-analyses on QST in both patients with CH and MH [32,33]. Indeed, some studies suggest that these descending pain modulation pathways could be altered in patients with CH [35,36,37,39,40,41] and MH [42,43,44]. Most of these alterations are evident during CH and MH attack periods, a finding that, combined with the alteration of descending pain modulation found at the neurophysiological level in CH [39,45] and the neuroimaging findings that will be described in this review, emphasizes the central mechanism’s crucial role in the development of CH and MH attacks [14]. However, a recent study showed no differences between MH and controls in conditioned pain modulation (CPM) and its related brain activity [46]. These findings highlight that, although central mechanisms play a key role in the pathogenesis of these primary headaches, different patient phenotypes may be present. This is clinically important, as it might affect patients’ treatment responses.

Although central sensitization is thought to be more involved in pain amplification and in the perpetuation of chronic MH than in episodic MH, some signs of central sensitization have been observed during and between attacks, suggesting that it could play a role both in its episodic and chronic forms of this condition [47].

Cutaneous allodynia is characterized by pain perception in response to normally non-painful stimuli applied to healthy skin [48]. It has been reported in 60% of MH patients and is even more common in those with chronic MH [49,50]. The development of cutaneous allodynia has been linked to the central sensitization of trigeminovascular neurons, and it is believed to be a manifestation of central sensitization during MH attacks [51]. A comparison of the resting-state connectivity between MH sufferers with and without cutaneous allodynia revealed differences in the connectivity of the PAG/nucleus cuneiformis with various pain-processing centers (brainstem, thalamus, insula, cerebellum) and higher-order pain-modulating regions (frontal and temporal areas) [52]. This finding is clinically relevant since it suggests that MH patients’ symptoms are associated with abnormal interictal communication between pain-modulating areas. Cutaneous allodynia can be assessed with QST [53] or with the Allodynia Symptom Checklist (ASC-12) [48], which has been translated and validated into Spanish, Turkish, Portuguese and German [54,55,56,57].

### 2.3. Neuroanatomy of the Trigemino-Autonomic Reflex and the Trigeminovascular System

As previously introduced, pain is often attributed to the activation of the trigeminovascular system in both MH and CH [58]. To delve deeper on this matter, it is worth noting that nociceptive fibers originating from the trigeminal ganglion extend to intracranial structures, including the dural, arachnoid, and pial blood vessels, as well as cerebral arteries and extracranial tissues [51,59]. From the trigeminal ganglion, nociceptive signals are transmitted to neurons within the trigeminocervical complex (TCC), which includes the trigeminal nucleus caudalis and the dorsal horn of the upper cervical spinal cord (C1-C2) [3]. Projections from the TCC terminate in the trigeminal brainstem nuclear complex, relaying somatosensory information through multiple pathways: to thalamic neurons via the trigemino-thalamic tract, to hypothalamic nuclei via the trigemino-hypothalamic tract, and to basal ganglia and brainstem nuclei, including the locus coeruleus and PAG [3,51,59]. These structures ultimately connect to various cortical regions responsible for processing nociceptive signals.

On the other hand, while the existence of neurotransmitters and neuropeptides related to postganglionic parasympathetic fibers (CGRP [60], nitric oxide synthase, VIP, and PACAP-38 [12]) is known, the results of two studies that attempted to investigate the exact mechanism of the trigemino-autonomic reflex suggest that peripheral activation, both afferent and efferent, is not sufficient to produce a CH attack [61,62]. Although the exact mechanisms leading to the activation of postganglionic parasympathetic fibers are unknown, the current paradigm suggests that central changes are responsible for activating parasympathetic efferents in some headache conditions [12].

In general, both parasympathetic peripheral mechanisms and changes related to the trigeminovascular system play a secondary role in the pathophysiology of CH and MH [3], with central mechanisms now considered the primary factors in the pathophysiology of the diseases [14,63].

In addition, as it will be discussed later, although neuroimaging studies have represented a significant advance in the scientific understanding of CH and MH pathogeneses, the exact central mechanisms, affected by a multifactorial involvement of different brain structures and neural networks, are still not fully understood [64].

**Figure 1:**
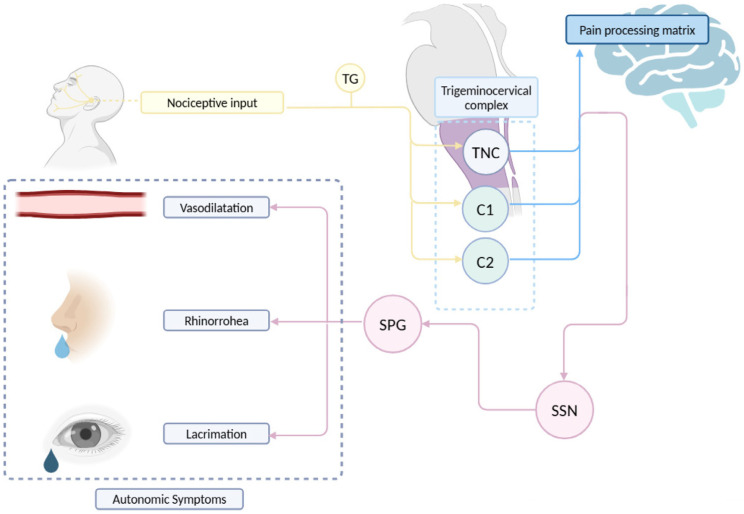
Schematic representation of the trigemino-autonomic reflex. TG: trigeminal ganglion. SPG: sphenopalatine ganglion. SSN: superior salivary nucleus. TNC: trigeminal nucleus caudalis. Edited with biorender.com.

The autonomic symptoms of CH are believed to be mediated by the trigemino-autonomic reflex (Figure 1). According to this model, nociceptive input traveling through the ophthalmic nerve during a CH attack can reflexively activate parasympathetic efferents [3]. Neuroanatomically, this is possible as the spinal trigeminal nucleus has connections with the superior salivatory nucleus (SSN), from which parasympathetic efferents of the greater petrosal nerve (a branch of the facial nerve) originate. These neurons synapse in the sphenopalatine ganglion [58]. The activation of these efferents, which innervate cranial vessels, explains the characteristic autonomic symptoms in CH (such as rhinorrhea, tearing or nasal congestion) and MH [3]. Nonetheless, in MH patients, it has been reported that these autonomic symptoms do not correlate with headache frequency or reversion to episodic frequency [65].

On the other hand, the trigeminal nerve provides sensory innervation to the extra- and intracerebral vessels, primarily via the ophthalmic nerve and the branches emerging from the trigeminal ganglion, forming the trigeminovascular system [66]. Nociceptive information then travels through Aδ and C fibers to synapse with second-order neurons in the spinal trigeminal nucleus (specifically the caudal subnucleus) and the upper cervical segments (C2-C3), targeting the superficial (I and II) and deep (V-VI) laminae [67,68]. Similarly, it has been demonstrated that stimulation of the trigeminal ganglion induces cerebral vasodilation in humans [69]. Therefore, in addition to being a major sensory relay for cerebral vascularization [70], the trigeminal nerve exhibits an efferent potential that could be relevant in the pathophysiology for headache conditions [71]. In Figure 1, the pathway of the trigeminal nerve, the projections of nociceptive information in the central nervous system, the connections between the nerve and the autonomic nervous system and the convergence in the trigeminocervical complex are schematically represented.

### 2.4. Genetics

A variant of the cryptochrome gene (CRY1) has been identified as significantly associated with the presence of CH, and this variant is expressed at elevated levels in these patients [72]. Additionally, genome-wide association studies have found several significant associations, suggesting that CH may be a disease characterized by genetic predisposition [73]. This was confirmed by a recent meta-analysis, which identified seven genetic risk loci associated with CH (DUSP10, MERTK, FTCDNL1, FHL5, WNT2, PLCE1, and LRP1), and also identified smoking as a causal factor in the disease’s etiology [74]. However, the attempts made so far to predict treatment effectiveness through genetic factors have not led to relevant findings [75]. Therefore, more genome-wide association studies are needed to highlight all the genetic variants that could explain genetic susceptibility and predict treatment response in patients with CH [76].

In contrast, genome-wide association studies have identified 123 risk loci associated with MH, especially in vascular and central nervous system tissue/cell types, supporting that neurovascular mechanisms which underlie MH pathophysiology [77]. Notably, certain genes, such as CALCA, CALCB, and HTR1F, have been implicated in MH pathophysiology and are targets for specific treatments [78]. Additionally, rare monogenic forms of MH, including familial hemiplegic MH, have been linked to mutations in genes like CACNA1A, ATP1A2, and SCN1A, which are involved in ion transport and neuronal excitability [76]. Also, studies reporting genetic factors related to patient’s prognosis have identified some polymorphisms associated with better outcomes for triptans [79,80]. Despite these advancements, the translation of genetic findings into predictive tools for treatment response remains limited. Further research is necessary to elucidate the full spectrum of genetic variants contributing to MH susceptibility and to develop personalized therapeutic strategies.

These findings highlight the importance of continued research into the genetic underpinnings of both MH and CH to better understand its pathophysiology and to develop more effective individualized treatments.

## 3. Neuroimaging in Cluster Headaches and Migraines

CH attacks typically follow a circadian and circannual rhythm. This clinical feature meant that research has identified the hypothalamus as a key center in CH pathophysiology. This stereotypical recurrence was first described more than half a century ago [81] and several decades later, early findings suggested a possible role of the hypothalamus in the pathogenesis of these diseases [82,83,84]. However, hypothalamic activation has also been found in other headaches, such as MH, both during the attack and before its onset [16,64,85], suggesting that it is not specific to CH or other trigemino-autonomic headaches. Although a circannual pattern has not been consistently described, MH shows also a cyclic nature [84]. Structural and functional neuroimaging studies have since identified alterations both at the hypothalamic and thalamic levels and more generally in structures related to pain processing in patients with CH and MH [14,64,86,87].

In CH, more than two decades ago, hypothalamic alterations were identified in the posteroinferior area ipsilateral to the pain, by inducing attacks with nitroglycerin and measuring subcortical activity using positron emission tomography [88], and by observing spontaneous attacks with magnetic resonance imaging [89]. Similarly, nitroglycerin has also been shown to provoke premonitory symptomatology associated with MH [90]. Additionally, structural alterations have been found in the anterior hypothalamic region, specifically in the suprachiasmatic nucleus, in patients with episodic and chronic CH, which may explain the stereotypical circadian and circannual rhythm of the attacks [90,91]. This nucleus is the main circadian pacemaker [92] and its alteration could contribute to the pathophysiology of CH. Some of the known mechanisms include biochemical processes related to the light–dark cycle processed by the retinohypothalamic tract through glutamate, PACAP-38 [93] and its subsequent secretion of melatonin [94], with levels being lower in CH patients [95].

Recent research has identified the anterosuperior subunit of the hypothalamus ipsilateral to pain as a key subcortical region in the pathophysiology of chronic CH [96]. This area contains the paraventricular nucleus and the preoptic area, which play roles in the modulation of trigeminovascular mechanisms and circadian regulation. Together with the strong association between the volume of this region and the number of daily attacks, this highlights its potential as a therapeutic target [96]. Although still hypothetical, the hypothalamus might play a central role in activating mechanisms related to the trigeminovascular system and the trigemino-autonomic reflex [12], as the hypothalamic paraventricular region has direct projections to the superior salivatory nucleus [97].

Moreover, the hypothalamus may alter the function of other interconnected regions, such as reducing its inhibitory capacity over trigemino-nociceptive neurons in the TCC [98]. Given that modifications in the functional connectivity of the hypothalamus with other regions have been identified, the hypothalamus does not appear to be the only subcortical center altered in the pathogenesis of these primary headaches. In fact, several alterations have been identified in structures related to pain processing.

By summarizing structural abnormalities that may be found in both headache conditions, it is worth noting that MH patients exhibit reduced grey matter in brain regions such as the frontal lobes, prefrontal cortex, left medial prefrontal cortex, brainstem, cerebellum, temporal lobes, right superior temporal, bilateral insula, cingulate cortex, orbitofrontal cortex, right occipital lobe, and right posterior parietal cortex [99,100,101]. Additionally, they may show reduced fractional anisotropy values in the superior and medial frontal lobes, as well as the right inferior frontal [100,101]. Cortical thickening is observed in the somatosensory cortex [102]. Functionally, MHs are characterized by enhanced activation in regions such as the anterior cingulate cortex [103], red nucleus, and substantia nigra [104], as well as stronger functional connectivity in various brain networks [105,106,107]

In contrast, CH patients exhibit reduced grey matter in the right thalamus, bilateral posterior hypothalamus, right posterior cingulate cortex, left inferior parietal lobe, head of the right caudate nucleus, bilateral middle frontal gyrus, right-middle temporal gyrus, right precentral gyrus, and left insula [108,109]. They also may demonstrate increased grey matter in the right cuneus [110], and exhibit grey matter volume changes in the temporal lobe, hippocampus, insular cortex, and cerebellum [111]. Cortical thinning is observed in the contralateral angular and precentral gyrus [111]. Functionally, CHs are characterized by enhanced activation in the posterior hypothalamus, anterior and posterior cingulate cortex, thalamus, basal ganglia, cerebellar hemispheres, prefrontal, insular, and temporal cortices [88,112], as well as altered functional connectivity in various central networks [113,114,115].

However, the two headache types also show some similarities in the involvement of subcortical structures that play a crucial role in pain descending modulation. The most distinctive magnetic resonance imaging (MRI) patterns for differentiating MH and CH patients from controls involved brain resting-state functional connectivity networks of the PAG and hypothalamus [116].

### Neural Networks and Pain Matrix

The hypothalamus plays a crucial role in not only in regulating endocrine and circadian rhythms but also as part of the pain matrix. It receives sensory information from the trigeminal nerve through the trigemino-hypothalamic tract and can modulate nociceptive neuronal activity ascending from the caudal subnucleus [117,118,119]. Indeed, it is widely recognized that MHs are associated with the activation and sensitization of trigeminovascular pathways, along with brainstem and diencephalic nuclei involvement [120]. In MH, dysfunctions in the brainstem, such as loss of habituation [87] or reduced cortical pre-activation [121] have been documented. Conversely, CH demonstrates altered pain perception and reduced pain thresholds [3].

Similarly, a recent study described cortical thinning in some regions involved in central pain processing in chronic CH patients (such as the medial cingulate cortex, posterior insula, and anterior cerebellar lobe) [122]. Additionally, there was an increase in functional connectivity of the ventral tegmental area, substantia nigra, subthalamic nucleus, red nucleus, and dorsal raphe nuclei with the ipsilateral hypothalamus in chronic CH patients [123]. Other researchers found greater functional connectivity in areas belonging to the default mode network in CH patients experiencing an acute attack, compared to patients free of attack, highlighting an increase in functional connectivity between the hypothalamus, anterior and posterior cingulate cortices, parahippocampal gyrus, and amygdala [124]. On the other hand, when making the same comparison, other studies found a decrease in connectivity between the hypothalamus and the medial frontal gyrus, precuneus of the parietal lobe, and cerebellum [125], as well as a reduction in the connectivity of the large-scale attentional network [126]. Taken together, these findings suggest that during an acute episode of CH, the hypothalamus connects less with brain areas/networks involved in pain inhibition, such as the cerebellum and frontal regions, resulting in a failure of pain modulation. In line with this, other authors found that patients with episodic CH during an attack had reduced activation of the tegmental area and salience network, which also translates into a diminished pain inhibition capacity [127]. Furthermore, during acute CH attacks, activation has been observed in other structures such as the thalamus, anterior cingulate cortex, and bilateral insula [89], which play a crucial role in the sensory-affective processing of the painful experience. Similarly, some authors have found an inverse relationship between the duration of CH and opioid receptor binding in the ipsilateral hypothalamus, pineal gland, and bilateral cingulate cortex [128], a finding that could also explain the altered pain modulation in CH patients. Additionally, resting-state functional magnetic resonance imaging data revealed significantly reduced functional connectivity between the frontal lobe and amygdala [129], which might suggest a failure in the inhibition of mesolimbic structures by prefrontal regions. Alterations were also found in auditory evoked potentials dependent on intensity during both attack and remission periods [130], which, by reflecting the bioavailability of serotonin at a central level, could suggest a reduction in the activity of the serotoninergic pathway from the raphe nuclei to the suprachiasmatic nucleus.

In MH, abnormalities have been observed in both ascending and descending nociceptive pathways during ictal and interictal phases [131]. Positron emission tomography (PET) studies have demonstrated increased activation of the dorsal pons in MH patients during the ictal phase [132]. Additionally, functional magnetic resonance imaging (fMRI) studies [107,133] have shown heightened functional connectivity between cortical and subcortical regions involved in nociceptive processing and the PAG, which receives inputs from the thalamus, hypothalamus, and autonomic nervous system. Specifically, the thalamus’ established role in both descending and ascending trigeminal pain processing and autonomic system regulation, could represent another sensitized subcortical center involved in altered pain processing, which could impair saliency detection mechanisms in the brain [134].

Another relevant hypothalamic system in nociception modulation is the orexinergic system, which regulates autonomic functions, wakefulness, and pain processing [119]. Research conducted in rats has proposed its potential role in the development of cortical spreading depression in MH and nociceptive activity related to the caudal portion of the trigeminal spinal nucleus [135,136]. In the same manner, it has also been suggested that there might be an alteration in the orexinergic system in CH patients [119]. These patients have lower levels of orexin-1 in cerebrospinal fluid [137], and a higher risk of presenting polymorphism in the orexin-2 gene [138,139].

Nonetheless, the most distinguishing MRI feature for differentiating migraine from cluster headache patients seems to be the left thalamic network, and hypothalamus resting state and functional connectivity. In comparison to migraine patients, those with CHs exhibited reduced functional connectivity between the left thalamus and cortical regions responsible for interoception and sensory integration [116].

## 4. Biomarkers in Cluster Headaches and Migraines

Studies conducted in animals have shown that experimentally induced nociceptive stimulation of the trigeminal system can lead to the release of vasodilatory molecules such as CGRP and vasoactive intestinal peptide (VIP) [140,141]. Similarly, this has been replicated in human studies when studying PACAP [142]. Indeed, biomarker research in these conditions mainly lies in animal studies. In that manner, CGRP, highly expressed in trigeminal ganglion neurons, plays a key role in the development of both primary headaches. It is released within the trigeminal ganglion and may contribute to peripheral sensitization by interacting with nearby neurons and satellite glial cells, ultimately facilitating central sensitization of second-order neurons [143]. In fact, CGRP is involved in the development of the attacks MH with aura [144], and it has also been experimentally reproduced in patients with acute CH during an attack and in patients with chronic CH, while this did not occur in patients with acute CH in remission phase [145]. Additionally, other authors have found similar results regarding PACAP-38, which, instead of presenting normalized levels in patients with episodic CH in remission, has been found to be decreased [146]. Although several studies have detected an increase in plasma levels of CGRP [147,148,149] and brain-derived neurotrophic factor (BDNF) [150] during CH and MH attacks, the possibility of inducing a CH attack by VIP and PACAP-38 without associated changes in plasma CGRP suggests that CGRP is not the only mediator of the attack [151]. Similarly, it has been shown that PACAP-38 infusion caused headache and vasodilatation in both healthy subjects and MH patients [152]. Moreover, BDNF represents another biomarker related to pain modulation and central sensitization, and it has been suggested to play a role in both MH and CH [150]. Two studies found that BDNF serum levels were altered in CH and MH [150,153]. CH patients showed significantly higher BDNF concentrations inside and outside cluster bouts compared with healthy controls. On the other hand, MH patients revealed significantly higher BDNF serum levels during migraine attacks compared with healthy controls, but no significant difference was found between MH with aura compared to those without aura, neither during MH acute attacks nor during headache-free periods [150].

Similarly, other biomarkers such as serotonin receptors (5-HT), endothelin-1 (ET-1) or substance P are increasingly recognized for their potential relevance in these conditions, despite not being strictly specific to either MH or CH. For instance, 5-HT may influence pain mechanisms, particularly through its association with neurogenic inflammation and modulation of vascular and neuronal pathways [116,154]. Moreover, the presence of ET-1, which is involved in vasoconstriction, might also play a role in headache pathophysiology [154].

The interrelationship of these biomarkers suggests a complex interplay where elevated levels of 5-HT, SP, and cytokines during headache episodes could reflect the wider neurovascular changes associated with these debilitating disorders. This convergence of findings implicates these markers as potential indicators of shared mechanisms alongside these conditions, warranting further investigation to definitively characterize their roles in diagnostics and treatment strategies for patients suffering from both headache types [116,155].

**Figure 2:**
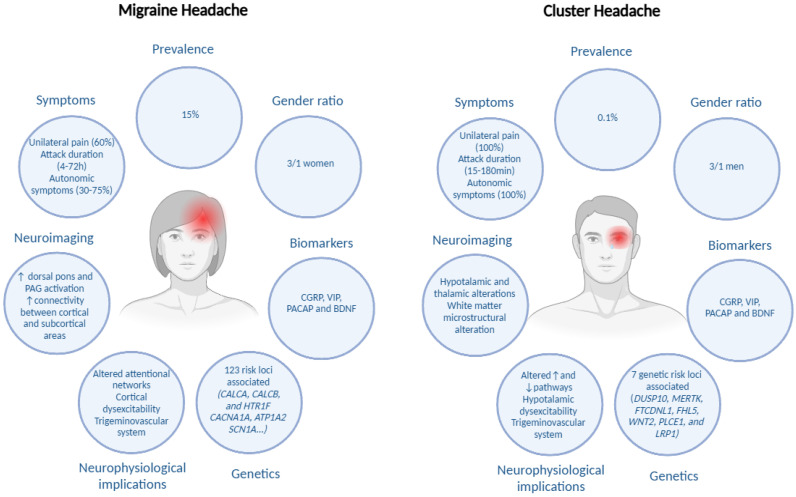
Summaries of shared and unique insights in cluster headache and in migraine headache. Edited with biorender.com.

However, the overall findings related to peripheral biomarkers present inconsistencies in line with those reported by a recent systematic review, as several biomarkers could represent potential markers of CH and MH activity, but they still do not differentiate patients with CH or MH from the healthy population or from other headaches [156]. Along with other characteristics of both conditions, Figure 2 shows the summarized main biomarkers in CH and MH.

## 5. Therapeutic Management of Cluster Headaches and Migraines

While biochemical alterations, such as the release of CGRP, PACAP, and VIP, have been well-documented in the pathophysiology of both CH and MH, these findings have also driven the development of novel therapeutic targets. These molecular insights have led to the creation of CGRP monoclonal antibodies (CGRPmAbs) and gepants, as well as the exploration of calcium channel blockers, which aim to modulate the underlying mechanisms of pain and vascular dysfunction. The only FDA-approved CGRP blocker for CH is Galcanezumab, which was found in a recent meta-analysis to be effective in the episodic form but not in the chronic form of CH [157]. However, other CGRP blockers have been shown to be ineffective in episodic CH (Eptinezumab) and chronic CH (Fremanezumab). On the other hand, CGRPmAbs and gepants (such as ubrogepant, rimegepant, and atogepant) were found to be effective as preventive treatment of MH in a recent meta-analysis [158].

Calcium channel-acting medications modulate neuronal excitability and vascular tone, making them useful in both CH and MH. Verapamil represents the first-line preventive treatment for CH (especially chronic CH) [9]. On the other hand, flunarizine is often used for migraine prevention in Europe, but its efficacy has recently been questioned in the light of the necessity to reevaluate its efficacy and establish more reliable data that meets the current standards of migraine treatment evaluation [159].

Despite the progress in understanding the biochemical pathways involved, there remains a need for further studies to refine and validate these treatments, ensuring they meet current clinical standards and provide effective outcomes for patients with primary headaches.

## 6. Limitations

This comprehensive review has several limitations. Firstly, authors tried to maintain an impartial perspective, but the intrinsic methodologic design may unintentionally introduce some risks of bias, such as selection and confirmation biases. In the same manner, this design does not allow for a proper analysis of a systematic methodological quality assessment of studies, which could estimate the potential risk of bias in different domains, as well as the publication bias which could also influence the concepts and ideas exposed in the present review.

## 7. Concluding Remarks

The understanding of CH and MH has progressed considerably, moving beyond the traditional view of these conditions as primarily vascular and nociceptive disorders. Contemporary research emphasizes the involvement of central neurophysiological mechanisms, including central sensitization, hypothalamic dysfunction, and alterations in neural networks. Although both conditions engage the trigeminovascular system, recent studies suggest that their underlying pathophysiology is more complex than previously thought. Advances in neuroimaging and genetics have provided valuable insights, yet many aspects of their mechanisms remain unclear. Table 1 shows a summary of the discussed insights during the manuscript regarding both conditions.

Despite these scientific developments, significant challenges remain in translating research findings into personalized treatment strategies. Current therapeutic approaches often target shared biological pathways, but the variability in individual patient phenotypes and their responses underscores the need for further investigation into predictive treatment targets.

In summary, CH and MH are complex neurological disorders requiring a multidisciplinary approach to enhance diagnosis, treatment, and patient care. Ongoing progress in neurophysiology, neuroimaging, and biomarkers will be essential for refining our understanding of these conditions and developing more effective and targeted therapies.

## Figures and Tables

**Table 1:** Summary of discussed insights in both conditions.

Characteristics	Cluster Headache	Migraine Headache
Pathophysiological Mechanisms	Activation of the trigeminovascular system. Unilateral pain is attributed more to central than peripheral changes. Vasodilation is considered an epiphenomenon, not a direct cause.	Activation of the trigeminovascular system. Functional changes between the hypothalamus, trigeminal nuclei, and dorsal pons may trigger attacks.
Central Mechanisms	Alterations in descending pain modulation observed during attacks. Studies suggest a pivotal role of central mechanisms in pathogenesis.	Central sensitization possibly contributes to pain amplification. Cutaneous allodynia is observed in 60% of cases, particularly in chronic migraine. Cerebral connectivity alterations reported.
Neuroanatomy	Activation of the trigemino-autonomic reflex (symptoms such as nasal congestion and tearing). Nociceptive fibers connect the TCC to structures like the superior salivatory nucleus and the sphenopalatine ganglion.	The trigeminovascular system plays a dual sensory and efferent role in pathophysiology. Trigeminal ganglion stimulation induces cerebral vasodilation.
Neuroimaging	Hypothalamus identified as a key center. Structural and functional alterations in nuclei such as the suprachiasmatic nucleus. Connectivity changes with the limbic system and prefrontal cortex during acute episodes.	Dorsal pons activation during attacks. Increased functional connectivity between cortical and subcortical regions. The orexinergic system may play a significant role.
Genetics	Up to 7 genetics associations have been identified. Smoking could be a causal factor.	123 risk loci identified. Genes related to rare monogenic forms, such as familial hemiplegic migraine. Some genetic variants may influence treatment response.
Hypothalamic Systems and Neural Networks	Hypothalamus linked to circadian regulation and pain. Increased functional connectivity with subcortical nuclei (tegmental area, raphe nuclei). Altered attentional networks during acute episodes.	Tracts such as the trigemino-hypothalamic pathway regulate nociception. The orexinergic system might influence cortical spreading and nociceptive processing in MH.
Biomarkers	CGRP released during acute and chronic attacks; PACAP-38 levels found decreased in episodic cluster headaches during remission. Elevated BDNF concentrations observed inside and outside cluster bouts.	CGRP and PACAP-38 induce pain and vasodilation. Elevated BDNF levels reported during attacks (no difference between with/without aura). Biomarkers do not yet guide treatment.
Therapeutic Options	CGRP blockers like Galcanezumab approved for episodic cluster headache (ineffective in chronic cases). Verapamil is the first-line preventive treatment for chronic cases.	CGRPmAbs and gepants reported to be effective. Flunarizine often used for prevention, but recent studies question its efficacy.

CRY1: Cryptochrome gene; CGRP: Calcitonin Gene-Related Peptide, PACAP-38: Pituitary Adenylate Cyclase-Activating Polypeptide; BDNF: Brain-Derived Neurotrophic Factor; mAbs: monoclonal antibodies.

## Data Availability

Not applicable.

## Abbreviations

The following abbreviations are used in this manuscript:

CH	Cluster Headache
ET-1	Endothelin-1
5-HT	Serotonin Receptors
MG	Migraine Headache
CGRP	Calcitonin Gene-Related Peptide
PAG	Periaqueductal Gray
QST	Quantitative Sensory Tests
TCC	Trigemino-Cervical Complex
